# A randomized controlled trial of nebulized surfactant for the treatment of severe COVID-19 in adults (COVSurf trial)

**DOI:** 10.1038/s41598-023-47672-x

**Published:** 2023-11-28

**Authors:** Ahilanandan Dushianthan, Howard W. Clark, David Brealey, Danny Pratt, James B. Fink, Jens Madsen, Helen Moyses, Lewis Matthews, Tracy Hussell, Ratko Djukanovic, Martin Feelisch, Anthony D. Postle, Michael P. W. Grocott

**Affiliations:** 1 Perioperative and Critical Care Theme, NIHR Southampton Biomedical Research Centre, University Hospital Southampton/University of Southampton, Southampton, UK; 2 General Intensive Care Unit, University of Southampton, University Hospital Southampton NHS Foundation Trust, Tremona Road, Southampton, SO16 6YD UK; 3 Clinical and Experimental Sciences, University of Southampton, Southampton, UK; 4 University College London Hospital, London, UK; 5 University College London Hospital Biomedical Research Centre, London, UK; 6 Elizabeth Garrett Anderson Institute for Women’s Health, University College London, London, UK; 7 Southampton NIHR Clinical Research Facility, University Hospital Southampton, Southampton, UK; 8 Aerogen Pharma Corporation, San Mateo, CA USA; 9 Lydia Becker Institute of Immunology and Inflammation, University of Manchester, Manchester, UK

**Keywords:** Microbiology, Medical research

## Abstract

SARS-CoV-2 directly targets alveolar epithelial cells and can lead to surfactant deficiency. Early reports suggested surfactant replacement may be effective in improving outcomes. The aim of the study to assess the feasibility and efficacy of nebulized surfactant in mechanically ventilated COVID-19 patients. Patients were randomly assigned to receive open-labelled bovine nebulized surfactant or control (ratio 3-surfactant: 2-control). This was an exploratory dose–response study starting with 1080 mg of surfactant delivered at 3 time points (0, 8 and 24 h). After completion of 10 patients, the dose was reduced to 540 mg, and the frequency of nebulization was increased to 5/6 time points (0, 12, 24, 36, 48, and an optional 72 h) on the advice of the Trial Steering Committee. The co-primary outcomes were improvement in oxygenation (change in PaO_2_/FiO_2_ ratio) and ventilation index at 48 h. 20 patients were recruited (12 surfactant and 8 controls). Demographic and clinical characteristics were similar between groups at presentation. Nebulized surfactant administration was feasible. There was no significant improvement in oxygenation at 48 h overall. There were also no differences in secondary outcomes or adverse events. Nebulized surfactant administration is feasible in mechanically ventilated patients with COVID-19 but did not improve measures of oxygenation or ventilation.

## Introduction

The Coronavirus Disease 2019 (COVID-19) pandemic caused by the SARS-CoV-2 virus has inflicted a significant health burden. As of September 2023, there were more than 770 million confirmed infections with 6.9 million deaths worldwide^1^. Despite significant advances in our understanding of the pathophysiological processes involved and improved therapeutic strategies, the mortality among mechanically ventilated patients with severe COVID-19 remains very high^2^. In patients with SARS-CoV-2 associated acute hypoxic respiratory failure requiring mechanical ventilation, effective therapeutic approaches are limited to standard supportive critical care, including ventilator bundles and prone positioning, and immunomodulator therapies (corticosteroids, anti-IL 6 or JAK inhibitors)^3^.

Pulmonary surfactant, synthesized and secreted by alveolar type II cells (AT-II), consists of a unique mixture of biomolecules (phospholipids, proteins, and cholesterol) and has a specific biophysical property that lowers alveolar surface tension during expiration^4^. Surfactant proteins, particularly SP-A and SP-D, are involved in the innate immune system with specific viral neutralizing functions^5^. Following cleavage of the transmembrane serine protease 2 (TMPRSS2), SARS-COV-2 binds to angiotensin-converting enzyme 2 (ACE2) receptors expressed on AT-II cells leading to epithelial cell dysfunction, apoptosis, and lack of precursors for the generation of type-1 cells^6–8^. Clinical and pathological features confirm similarities between COVID-19 and surfactant-deficient neonatal respiratory distress syndrome^9,10^. Moreover, there is evidence of altered surfactant synthesis and composition in COVID-19^11–13^. Although studies of exogenous surfactants in adult patients with acute respiratory distress syndrome (ARDS) have not shown any mortality benefits^14^, they were limited by patient heterogeneity and variations in dosage, delivery methods, and surfactant preparations, precluding any definitive conclusions^15,16^.

Effective exogenous surfactant delivery to improve the alveolar epithelial barrier function remains challenging in adults. Previous replacement studies of nebulized surfactant in ARDS were limited by poor alveolar deposition^17^. Recent advances in nebulizer technology utilising novel vibrating mesh combined with breath-synchronised delivery during the inspiratory phase enable considerable improvements in alveolar surfactant delivery^18^. In a porcine surfactant-deficient model, breath-synchronised nebulized surfactant therapy improved surfactant delivery and oxygenation and prevented the development of inflammatory lung injury with doses of 540–1080 mg surfactant^19^. Using this novel delivery method, the objective of this study is to assess the feasibility, efficacy, and safety of a nebulized natural bovine surfactant in mechanically ventilated patients with severe COVID-19 pneumonia.

## Methods

### Study design

This is a pilot, exploratory, dose-adaptive, prospective, randomized, phase-2, open-label, proof-of-concept trial to assess the feasibility, safety, and efficacy of nebulized surfactant (Alveofact®, bovactant) in adult COVID-19 patients requiring mechanical ventilation (NCT04362059, date of registration 24/04/2020). The trial was conducted in the intensive care units at University Hospital Southampton (UK) and University College Hospitals London (UK)^20^. The study was sponsored by University Hospital Southampton (RHM CRI0399) and approved by the Health Research Authority (HRA), UK and Health Care Research Wales Ethics Committee (20/NE/0149), IRAS ID: 282498. Informed consent was obtained from all subjects and/or their legal representatives. The trial conducted in accordance with Good Clinical Practice (GCP), Health Research Authority (HRA) and Medicines and Health Care Regulatory Agency (MHRA, UK) regulations and standards. The reporting of this manuscript adheres to CONSORT guidelines. The study protocol is available as a supplementary file.

**Figure 1:**
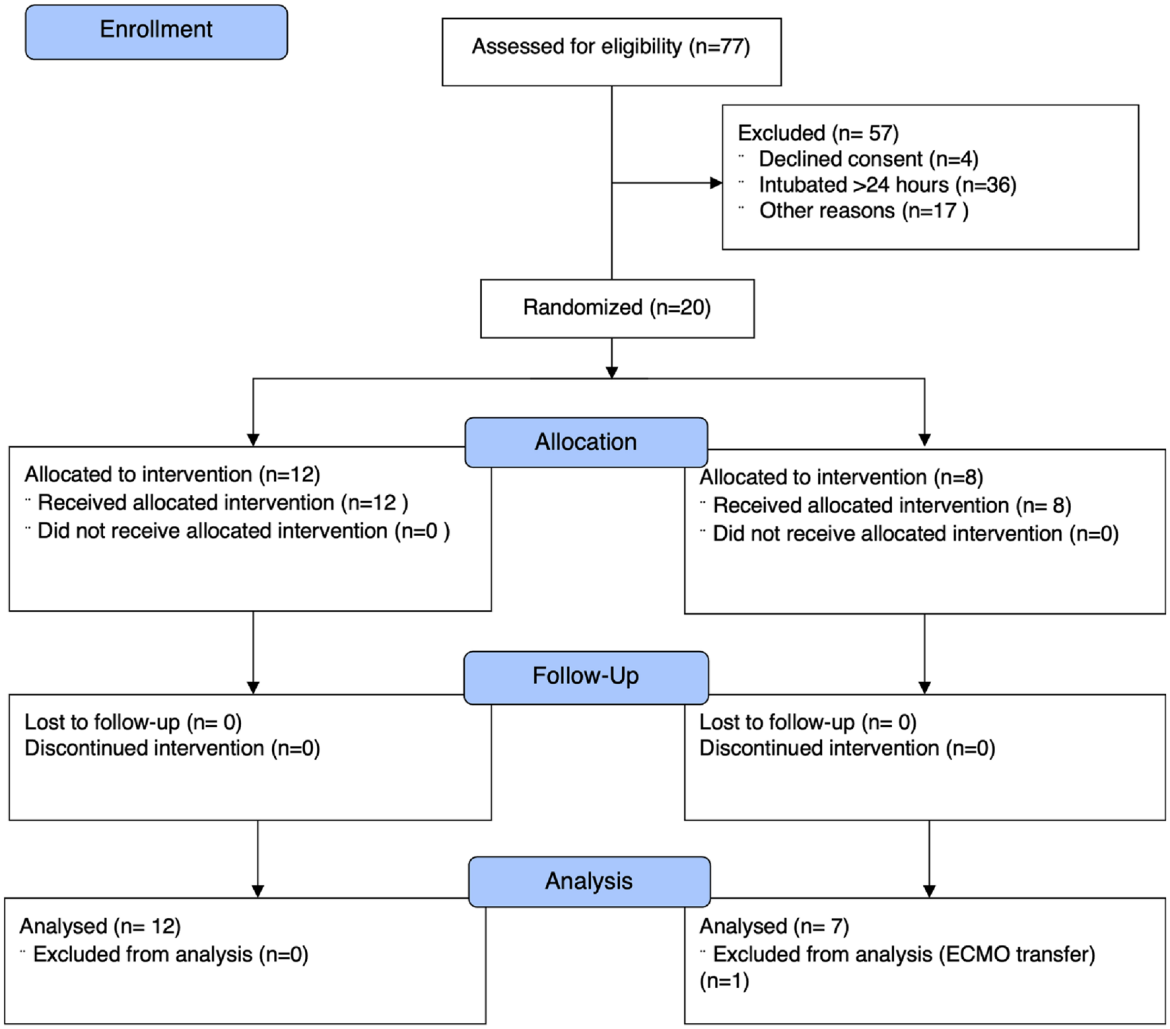
Consort diagram for enrolment and randomization.

Eligible participants were 18 years of age or older, admitted to the intensive care unit and requiring invasive mechanical ventilation for acute respiratory failure following a positive respiratory sample real-time polymerase chain reaction (RT-PCR) SARS-CoV-2 test. All patients had radiological evidence of SARS-CoV-2 viral pneumonia and enrolled within 24 h of endotracheal intubation. The study was conducted between October 2020 and December 2021. Between 14th of October 2020 and 12th of November 2021, 77 patients were assessed for eligibility, and 20 patients were randomized. The University Hospital Southampton and University College Hospital London enrolled 13 and 7 participants respectively. One patient from the control group was transferred to the regional Extracorporeal Membrane oxygenation (ECMO) centre for additional support before study assessment at 48 h, leaving 19 participants for the primary analysis (Fig. 1).

### Trial procedures

Participants were randomly assigned to receive open-label nebulized surfactant or no intervention in a 3:2 ratio using an internet-based block randomization service (ALEA tool for clinical trials, FormsVision BV). As patients were unable to consent, consent was obtained from a personal legal representative (PerLR) or professional legal representative (ProfLR) prior to randomisation. Once regained capacity, all participants were approached for their consent for continued participation. The trial steering committee designed the protocol, and the oversight was provided by independent data and safety monitoring board (DSMB) and an external contract research organization (PHARMExcel). The trial protocol was previously published and registered in ClinicalTrials.gov (NCT04362059). All relevant data were collected and analyzed by the investigators. All the authors assume responsibility for the accuracy and completeness of trial data and the trial fidelity to the protocol and statistical analysis plan.

### Surfactant composition

The recruiting centre did randomisation with a unique subject identifier specific to that centre. The nebulized surfactant is a natural lyophilized bovine surfactant (Alveofact®) preparation with an approximate composition of phospholipids [phosphatidylcholine (75%), phosphatidylglycerol (13%), phosphatidylethanolamine (3%), phosphatidylinositol (1%) and sphingomyelin (1%)], cholesterol (5%), surfactant proteins (1% SP-B and SP-C), and very low levels of free fatty acids, lysophosphatidylcholine, water, and 0.13% calcium. Each Alveofact® lyophilized vial consists of 108 mg of surfactant mixed at the bedside with a prefilled syringe containing 2.2 ml of 0.45% saline buffer for administration into the nebulizer.

### Nebulizer device

**Figure 2:**
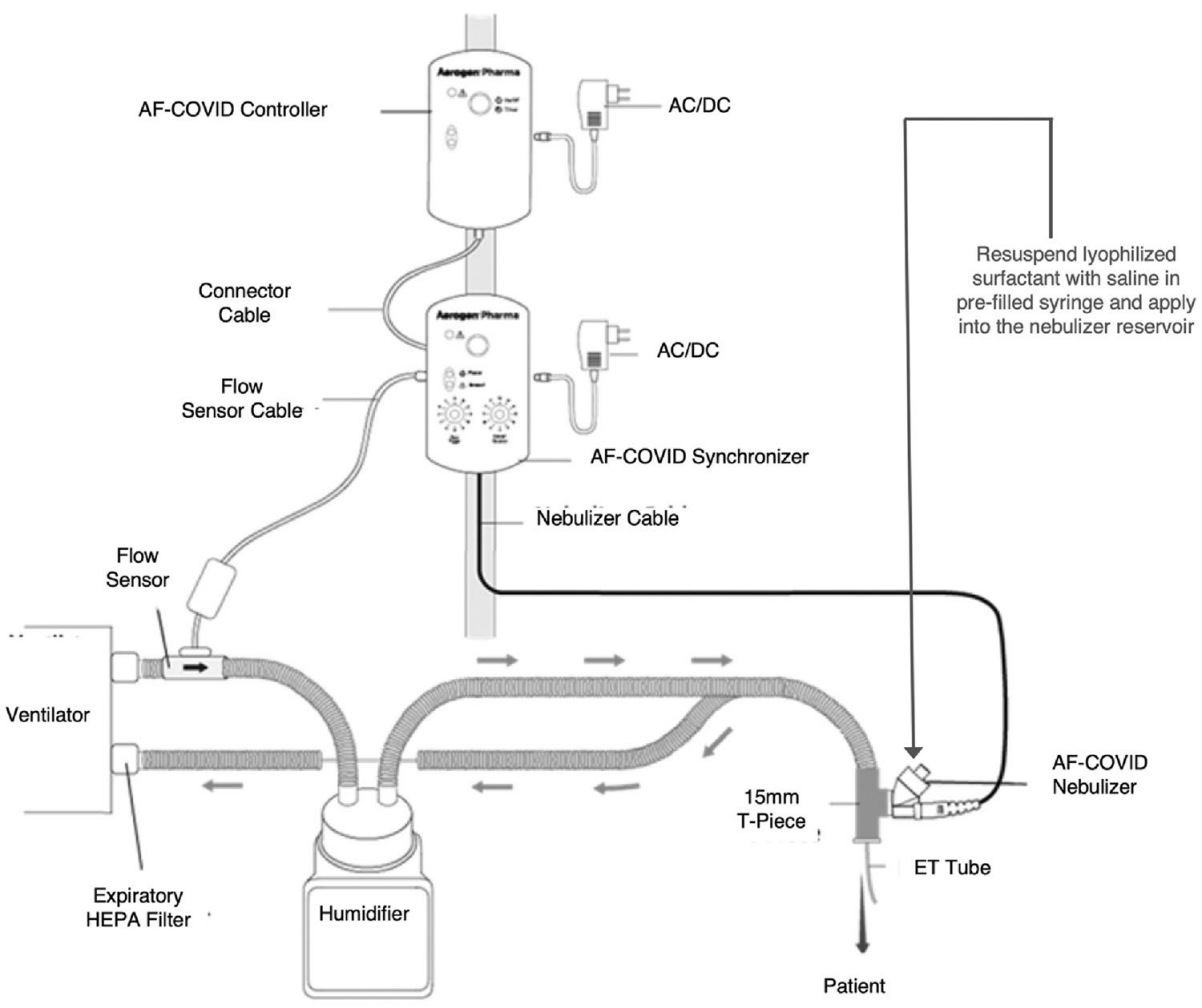
The nebulizer device and the ventilator circuit connections with the controller and breath synchronizer (modified from reference 19).

This nebulizer device (Aerogen, Galway, Ireland) has a novel two-layer photo defined aperture plate (PDAP) vibrating mesh generating tiny surfactant droplets with mass median aerodynamic diameter < 3 µm to enhance distal lung deposition^19^. The nebulizer is controlled by two pole-mounted controllers with a sensor attached to the inspiratory limb of the ventilator to sense the start of the inspiratory phase and spray time adjusted to nebulize surfactant during the first 80% of the inspiratory phase of delivered breaths to optimize delivery and minimize wastage. The nebulizer is positioned between the ventilator circuit and the endotracheal tube (Fig. 2).

### Nebulization dosing and timing

**Figure 3:**
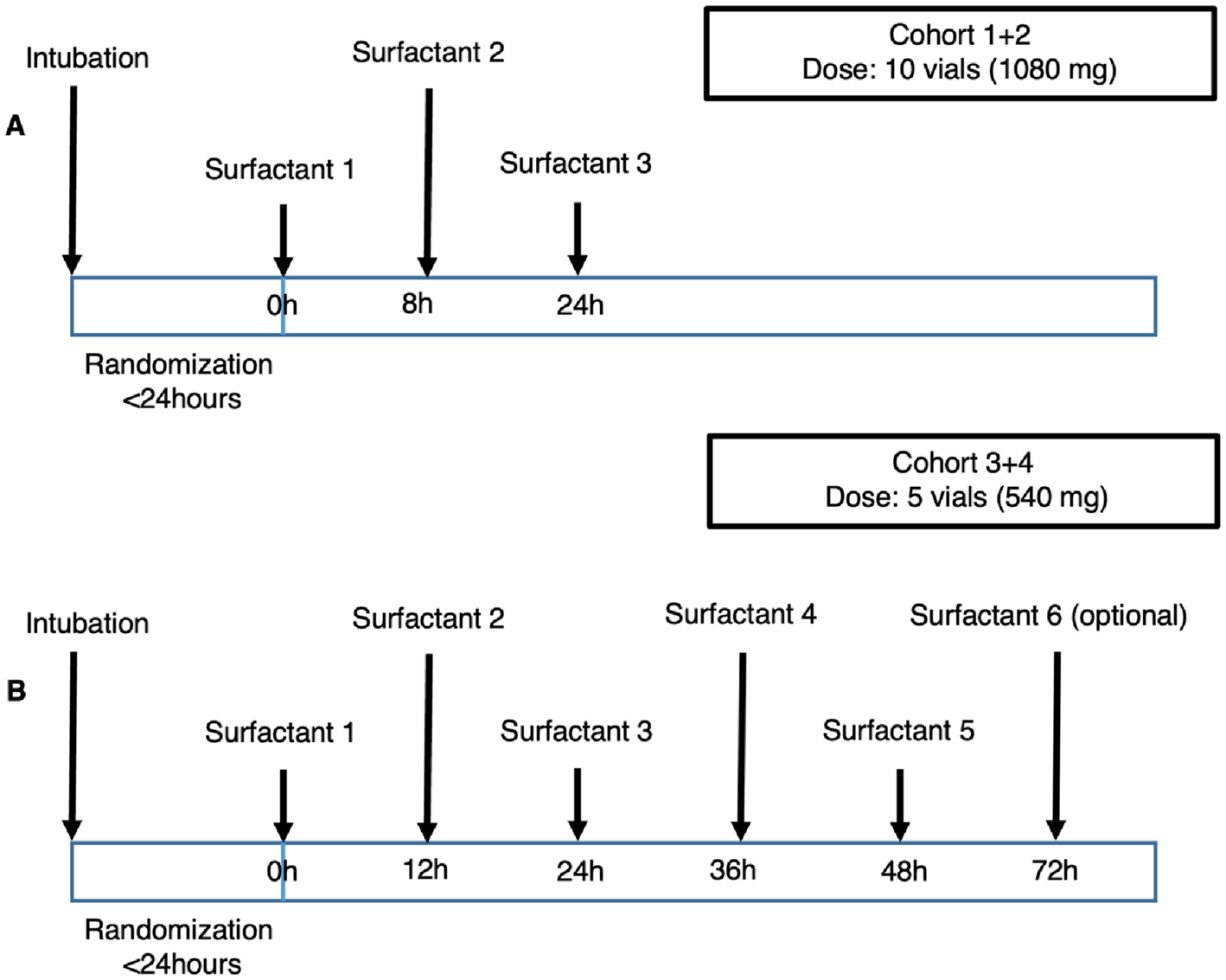
Surfactant dosing regimen.

For the first 10-patient cohort, surfactant was administered at 0, 8, and 24-h post-randomization (Fig. 3). Due to the exploratory dose-finding nature of this study, the trial participants were divided into four cohorts with prospective graded dose escalation of total surfactant dose from 30 to 90 vials (3240–9720 mg). The first cohort received ten vials (1080 mg) at each dose scheduled at 0, 8 and 24 h. However, each vial took around 10–15 min to deliver, making it practically impossible to escalate the dose any further. The trial Data and Safety Monitoring Board (DSMB) was convened after this first cohort (N = 5) and advised the Trial Steering Committee (TSC) to proceed with the current dosing schedule for the second cohort, which was approved. Following completion of the second cohort (N = 5), biochemical analyses were conducted from the endotracheal aspirates, which confirmed an effective delivery but a rapid turnover with an estimated half-life for Alveofact phospholipid of ~ 7.6 h (range 1.8–20.8 h)^11^. Following further review by the DSMB and approval by the TSC, the dosing schedule was modified to provide a more frequent and prolonged surfactant administration with altered delivery timings as 0, 12, 24, 36, 48 h, and an optional dose at 72 h after randomization. In addition, the dose delivered was reduced from 1080 mg (10 vials) to 540 mg (5 vials). This dosing regime was maintained for cohorts 3 and 4 (Fig. 3).

### Exclusion criteria

The exclusion criteria were imminent expected death within 24 h, specific contraindications to surfactant administration (e.g. known allergy, pneumothorax, pulmonary haemorrhage), known or suspected pregnancy, stage 4 chronic kidney disease or requiring dialysis (i.e., estimated glomerular filtration rate < 30 ml/min), liver failure (Child–Pugh Class C), anticipated transfer to another hospital within 72 h (which is not a study site); current or recent (within 1 month) participation in another study that, in the opinion of the investigator, would prevent enrolment for safety reasons, and declined consent or assent. Patients were ineligible if they were intubated for more than 24 h.

### Trial outcomes

The co-primary outcome was an improvement in oxygenation (PaO_2_/FiO_2_ ratio) and pulmonary ventilation defined as Ventilation Index (VI), where VI = [RR × PiP × PaCO_2_]/1000 at 48 h after study initiation. Ventilation index has been used widely by the paediatric surfactant replacement studies and provides a measure of the ventilation and incorporates variables from PaCO_2_, peak airway pressure and respiratory rate^21–23^. The secondary outcomes include frequency and severity of adverse events, change in pulmonary compliance, positive end-expiratory pressure (PEEP), ventilation index and PaO_2_/FiO_2_ ratio at 24 h after study initiation, clinical improvement defined by time to one improvement point on the ordinal scale described in the WHO master protocol (2020) recorded while hospitalised, duration of mechanical ventilation, mechanical ventilator-free days (VFD) at day 21, length of intensive care unit stay, number of days hospitalised and mortality at day 28.

### Surfactant phosphatidylcholine assessment

Endotracheal aspirates through a closed in-line tracheal suction system were taken from all patients before surfactant nebulization and at 8, 16, 24, 48 and 72 h after recruitment. Samples were filtered and centrifuged at 400 g for 10 min at 4 °C. The supernatant was then lipid extracted by a modified Bligh and Dyer method^24^. Lipid extracts were analysed by electrospray ionisation mass spectrometry (ESI–MS/MS) (Waters Corporation, UK) to quantify surfactant phospholipids^25,26^.

### Adverse event reporting

COVID-19 participants enrolled in this study were already critically ill with multiple COVID-19 related medical issues and were at risk of ongoing clinical deterioration and multi-organ failure. It was expected that many of these patients would experience events during their clinical pathway, but these were not reported unless the event was considered by the investigator to be associated with the study drug or delivery.

Any of the following pre-specified respiratory and cardiovascular deteriorations or adverse events occurring during nebulization and occurring within 48 h were recorded.

1. Increase in oxygen (FiO_2_ ≥ 0.2 or more) or ventilator requirements (increase in PEEP of > 5 cmH_2_O or more to maintain target oxygenation).
2. Sustained deterioration in pulmonary ventilation variables (> 10% increase in peak or mean airway pressures or decrease in tidal volume).
3. Any episode of new cardiac arrhythmia
4. Sustained increase in heart rate of > 20%
5. Sustained reduction in mean arterial blood pressure (MAP) of > 10% or an increase in the vasopressor dose of Norepinephrine (0.1mcg/kg/min), epinephrine (0.1 mcg/kg/min) or the use of additional inotropes (dopamine/dobutamine/milrinone) or vasopressors (vasopressin/terlipressin/phenylephrine).
6. New bronchospasm requiring treatment.
7. Other respiratory deteriorations: pneumothorax (evidence on imaging), pulmonary haemorrhage (clinical) and acute lobar collapse (evidence on imaging), including transfer to tertiary hospital for ECMO were collected.

### Statistical analysis

This is a pilot, exploratory dose-adoptive study and power calculations were based on significant dose response under varying assumed true dose response when using matched controls. Baseline data are presented as means (standard deviation) or medians (interquartile range) depending on the Normality of distribution. Categorical and binary variables are summarised as frequency and percentage of total. Baseline characteristics were compared between groups using independent samples t-tests for means, Mann–Whitney-U test for medians, and Fisher’s exact test for categorical data. Significance was defined at *p* < 0.05. For the co-primary endpoints of change in PaO_2_/FiO_2_ ratio and ventilation index, the difference between baseline and 48 h was calculated, and the Wilcoxon–Mann–Whitney test was used to test for the difference between groups. We then used quantile regression to adjust for the baseline value of the outcome variable. We also performed a secondary analysis of the primary endpoint splitting the surfactant group into cohorts 1&2 and cohorts 3&4, using the Kruskal Wallis test to investigate difference between the three groups (overall, cohorts 1&2, cohorts 3&4). The number of adverse events and their relationship to the study treatment were summarised as frequency and percentage of total, and risk ratios are reported with 95% confidence intervals. Surfactant phosphatidylcholine measurements are presented as percentage of total phospholipid as mean values and standard error of mean.

## Results

Baseline characteristics were similar between surfactant and control groups (Table 1). Mean participant age was 55.5 versus 56.5 years, and women accounted for 42% and 38% respectively. Median body mass index was ≥ 30 kg/m^2^ for both groups and clinical frailty scores were similar. The presence of comorbidities including diabetes mellitus, hypertension, ischaemic heart disease, chronic respiratory and kidney disease were also similar. Although demographic and chronic health characteristics were similar between groups, severity of illness measures tended to be higher in the surfactant group including APACHE II scores (15.6 vs. 11.0, *p* = 181), SOFA scores (6.1 vs. 5.1, *p* = 0.280), CRP (129 vs. 48, *p* = 0.091) and neutrophil lymphocyte ratios (N/L 17.0 vs. 9.5, *p* = 0.115). Baseline ventilation indices and oxygenation quantified by the PaO_2_/FiO_2_ were similar between groups. There were significant between-group differences in the laboratory markers, creatinine and creatine kinase at baseline. There were no significant differences between groups for the interventions received in ICU. Although not statistically significant, the surfactant group had shorter hospitalization prior to recruitment (93 h vs. 194 h, *p* = 0.177) and shorter period spent on non-invasive respiratory support (high flow nasal oxygen, continues positive airway pressure or non-invasive ventilation) prior to initiation of mechanical ventilation. The oxygenation status just prior to intubation was not different between groups (Table 1).

**Table 1:** Baseline characteristics of all recruited patients.

Baseline characteristics	All patients n = 20	Controls n = 8	Surfactant n = 12	*p* value*
Age-yrs	55.9 (12.7)	56.5 (11.4)	55.5 (14.0)	0.869
Female sex-n (%)	8 (40%)	3 (38%)	5 (42%)	0.852
Ethnicity				
White	16 (80%)	7 (88%)	9 (75%)	0.470
Mixed	2 (10%)	0 (0%)	2 (17%)	
Asian	2 (10%)	1 (13%)	1 (8%)	
Body mass index (kg/m^2^)	32.3 (28.0, 38.8)	30.8 (27.8, 42.0)	33.5 (28.0, 38.8)	1.000
Clinical frailty score	2 (2, 3)	2 (1, 2.5)	2 (2, 3)	0.333
Pre-existing conditions				
Diabetes mellitus	7 (35%)	3 (38%)	4 (33%)	0.921
Hypertension	3 (15%)	2 (25%)	1 (8%)	0.306
Ischaemic heart disease	3 (15%)	1 (13%)	2 (17%)	0.735
Chronic heart failure	0 (0%)	0 (0%)	0 (0%)	N/A
Chronic respiratory disease	3 (15%)	2 (25%)	1 (8%)	0.523
Chronic kidney disease	2 (10%)	2 (25%)	0 (0%)	0.147
Cancer (active or treatments < 1 year)	0 (0%)	0 (0%)	0 (0%)	N/A
Chronic liver disease	0 (0%)	0 (0%)	0 (0%)	N/A
Immunosuppression	0 (0%)	0 (0%)	0 (0%)	N/A
APACHE II score	13.8 (7.4)	11.0 (8.8)	15.6 (6.0)	0.181
SOFA score	5.7 (3.1)	5.1 (3.8)	6.1 (2.6)	0.280
Ventilation and oxygen parameters				
PaO_2_ (mmHg)	66.8 (60.8, 75.5)	64.5 (60.8, 69.8)	67.5 (61.5, 81.8)	0.460
FiO_2_ (%)	0.6 (0.6, 0.8)	0.7 (0.5, 0.9)	0.6 (0.6, 0.8)	0.815
PaO_2_/FiO_2_ (mmHg)	118.1 (40.6)	115.8 (43.1)	119.7 (40.8)	0.842
Respiratory rate	17.9 (4.1)	16.9 (4.9)	18.5 (3.6)	0.401
Compliance (cmH_2_O)	39.8 (14.8)	36.0 (12.7)	42.1 (16.1)	0.443
PEEP (cmH_2_O)	12.0 (8.5,12.5)	12.0 (5.0,13.0)	11.0 (8.5,12.5)	0.833
Tidal volumes (ml)	453 (413, 499)	458 (425, 474)	444 (382, 533)	0.777
Tidal volumes (ml/PBW)	7.1 (6.1, 8.0)	6.7 (6.1,7.1)	7.6 (6.4, 8.1)	0.187
Peak pressures (cmH_2_O)	26.7 (4.6)	29.0 (3.6)	25.1 (4.7)	0.061
Plateau pressure (cmH_2_O)	26.1 (4.8)	28.0 (4.6)	24.8 (4.7)	0.222
Admission laboratory results				
Alanine Aminotransferase (IU/l)	42.0 (25.0, 75.5)	33.5 (23.0, 174.0)	44.5 (27.0, 72.0)	0.895
Bilirubin (μmol/l)	10.1 (4.0)	12.1 (4.2)	8.7 (3.4)	0.070
Creatinine (mmol/l)	63.0 (48.0, 81.0)	73.5 (62.0, 123.5)	51.5 (38.0, 70.5)	0.047†
Creatine kinase (U/l)	102 (35.0, 518.0)	35.0 (27.5, 62.5)	518 (315.0, 62.5)	0.029†
C-reactive protein (mg/l)	101 (17.0, 158.0)	48.0 (9.5, 114.3)	129 (21, 198)	0.091
Ferritin (mg/l)	774 (405,1510)	1152 (680, 1510)	708 (306, 922)	0.371
Lactate dehydrogenase (U/l)	688 (436, 1361)	636 (628, 1361)	721 (436, 872)	0.898
Neutrophil/lymphocyte ratio	12.3 (6.8, 23.4)	9.5 (6.4, 13.1)	17.0 (10.2, 24.5)	0.115
Platelet count (× 10^9^/l)	337.5 (117.2)	301.4 (132.0)	358.6 (105.8)	0.297
Urea (mmol/l)	7.3 (5.0, 12.3)	9.5 (5.4, 15.4)	6.4 (5.0, 7.8)	0.135
ICU interventions				
Prone positioning, n (%)	17 (85%)	8 (100%)	9 (75%)	0.242
Dexamethasone, n (%)	20 (100%)	8 (100%)	12 (100%)	1.000
Tocilizumab, n (%)	7 (35%)	2 (25%)	5 (41.7%)	0.642
Nitric oxide, n (%)	8 (40%)	4 (50%)	4 (33.3%)	0.648
Cisatracurium, n (%)	15 (75%)	6 (75%)	9 (75%)	1.000
Antibiotics, n (%)	20 (100%	8 (100%)	12 (100%)	1.000
Remdesivir, n (%)	5 (25%)	1 (12.5%)	4 (33.3%)	0.602
Vasopressors (any), n (%)	18 (90%)	7 (87.5%)	11 (91.7%)	1.000
Respiratory support prior to mechanical ventilation				
Duration hospitalization prior to study recruitment, (hours)	107 (69, 221)	194 (126, 244)	93 (64, 125)	0.177
Proportion with NIRS support prior to IMV, n (%)	20 (100%)	8 (100%)	12 (100%)	1.000
Duration of NIRS support prior to MV (Hours)	59 (28, 115)	118 (47, 168)	43 (17, 81)	0.059
FiO_2_ requirement pre-IMV (%)	0.85 (0.75, 0.90)	0.78 (0.74, 0.86)	0.85 (0.75, 0.90)	0.509
PaO_2_/FiO_2_ pre-IMV (mmHg)	78.0 (73.0, 91.1)	79.5 (77.1, 86.6)	76.7 (71.7, 91.1)	0.562

*APACHE II* acute physiology and chronic health evaluation II score, *FiO*_*2*_ fractional inspired oxygen, *IMV* invasive mechanical ventilation, *NIRS* non-invasive respiratory support, *HFNO* includes high flow nasal oxygen, *CPAP* continuous positive airway pressure, *NIV* non-invasive ventilation, *PaO*_*2*_ partial pressure of oxygen in arterial blood (mmHg), *PEEP* positive end expiratory pressure, *SOFA* sequential organ failure assessment score.

Data presented as mean and standard deviation, median and interquartile range or numbers (%).

*Two-sample t-test for means, Mann–Whitney-U test for medians, and Fisher’s exact test for categorical data.

^†^Significant differences (*p* < 0.05) within Mann–Whitney-U test.

### Feasibility outcomes

All patients received all prescribed doses of surfactant. There were two episodes of delays in surfactant delivery due to issues relating to the device, one related to failure of breath synchronisation and one to breath-sensor failure. The devices were replaced immediately on both occasions and patients subsequently received their appropriate dose. No other device related issues were documented. The first two cohorts (cohort 1 + 2) had 10 surfactant vials delivered with a median nebulizer delivery time of 252 min (IQR 190, 283). The next two cohorts (cohort 3 + 4) had a reduced surfactant dose (5 vials) with a much shorter median nebulization duration of 87 min (IQR 70, 98).

### Efficacy outcomes

#### Co-primary outcomes

There was no difference in the primary outcomes (change in PaO_2_/FiO_2_ and ventilation index at 48 h) between groups in the unadjusted or baseline adjusted analyses. Median change in PaO_2_/FiO_2_ ratio from baseline to 48 h was 25.0 mmHg (IQR − 43.0 to 108.9) for the surfactant group and 54.2 mmHg (IQR − 40.9 to 71.2) for the control group. Median change in ventilation index was 0.1 (IQR − 3.8 to 2.9) for the surfactant group and 1.4 (IQR − 3.3 to 14.1) for the control group (Table 2).

**Table 2:** PaO_2_/FiO_2_ ratio and ventilation index at baseline and 48-h after randomization and the change from baseline.

Outcome	Control N = 7	Surfactant (all) N = 12	*p* value*
PaO_2_/FiO_2_ ratio			
Baseline	119.5 (74.5, 145.9)	111.3 (102.1, 134.8)	
48 h	139.1 (90.0, 186.4)	153.7 (83.0, 200.4)	
Change baseline to 48 h	54.2 (− 40.9, 71.2)	25.0 (− 43.0, 108.9)	0.77
Ventilation Index			
Baseline	22.6 (20.0, 25.4)	20.0 (17.5, 27.1)	
48 h	25.9 (19.5, 29.8)	19.8 (17.0, 33.3)	
Change baseline to 48 h	1.4 (− 3.3, 14.1)	0.1 (− 3.8, 2.9)	0.77

*FiO*_*2*_ fractional inspired oxygen, *PaO*_*2*_ partial pressure of oxygen in arterial blood (mmHg).

Data presented as median and interquartile range.

*Wilcoxon–Mann–Whitney test.

### Secondary outcomes

There were no differences in PaO_2_/FiO_2_ ratio at 24 h, ventilation index at 24 h, pulmonary compliance at 24 and 48 h or PEEP requirement at 24 and 48 h between the surfactant treated group and the controls. There were no differences in other secondary outcomes including change in World Health Organisation (WHO) Ordinal Scale, duration of mechanical ventilation, length of ICU stays, and number of days hospitalised. Four patients from the surfactant group and one from the control group had died by day 28. There were no between-group differences for the secondary outcomes of the two surfactant dosage groups (Cohort 1 + 2 vs. Cohort 3 + 4; Table 3).

**Table 3:** Secondary outcomes.

Secondary outcome measures	Controls N = 7	Surfactant N = 12	*p* value*
Change in PaO_2_/FiO_2_ ratio at 24 h (mmHg)	18.1 (38.0)	23.5 (54.0)	0.82
Change in VI at 24 h	0.0 (− 3.3,12.9)	− 1.5 (− 3.6,1.1)	0.37
Change in pulmonary compliance (cmH_2_O)			
24 h	− 6.2 (11.5), n = 5	0.5 (10.6), n = 10	0.28
48 h	− 0.8 (10.0), n = 4	− 2.1 (9.1), n = 8	0.82
Change in PEEP (cmH_2_O)			
24 h	0.0 (0.0, 0.0)	0.0 (− 0.5, 0.0)	0.83
48 h	0.0 (0.0, 12.0)	0.0 (0.0, 0.0)	0.21
WHO Ordinal Scale			
Showed any improvement	4 (50%)	5 (42%)	0.54
Time (days) to one improvement point	11 (10, 12)	13 (11, 16)	0.48
Duration of mechanical ventilation (days)	12 (10, 28)	15 (10, 28), n = 9	0.89
Ventilator-free days at Day-21	4.5 (0, 11)	0 (0, 9), n = 11	0.52
Length of ICU stay (days)			
All	27 (19.5, 48)	20.5 (13, 32.5)	0.28
Survivors	23 (18, 58)	27.5 (15, 48.5)	0.75
Number of days hospitalised			
All	35 (27.5, 63)	33 (20, 52)	0.53
Survivors	38 (27, 74)	39 (25.5, 60)	0.93
Mortality at 28 days	1 (12.5%)	4 (33%)	0.60

*FiO*_*2*_ fractional inspired oxygen, *ICU* intensive care unit, *PaO*_*2*_ partial pressure of oxygen in arterial blood (mmHg), *PEEP* positive end expiratory pressure, *VI*  ventilation index, *WHO* World Health Organization.

Data are presented as median and interquartile ranges or number (%).

*Two-sample t-test for means, Mann–Whitney-U test for medians, and Fisher’s exact test for categorical data.

### Sub-group analysis for the primary outcome

There were no between-group differences for the primary outcomes of the two surfactant dosage groups (Cohort 1 + 2 vs. Cohort 3 + 4; Table 4).

**Table 4:** Primary outcome according to the two different surfactant groups.

Outcome	Control N = 7	Surfactant 1 (Cohorts 1 + 2) N = 6	Surfactant 2 (Cohorts 3 + 4) N = 6	*p* value*
PaO_2_/FiO_2_				
Baseline	119.46 (74.55, 45.87)	118.21 (72.21, 150.01)	107.31 (102.26, 122.14)	
At 48 h	139.10 (90.01, 186.39)	153.68 (83.21, 182.51)	157.66 (76.68, 206.80)	0.73
Change baseline to 48 h	54.17 (− 40.91, 71.15)	20.80 (− 58.43, 114.75)	55.53 (− 40.55, 103.06)	
Ventilation index				
Baseline	22.6 (20.0, 25.4)	23.9 (15.0, 35.1)	19.8 (17.5, 22.5)	
At 48 h	25.9 (19.5, 29.8)	19.8 (12.3, 33.0)	19.9 (19.6, 42.4)	
Change baseline to 48 h	1.4 (− 3.3, 14.1)	− 2.0 (− 2.7, 1.8)	2.3 (− 4.9, 20.7)	0.56

*FiO*_*2*_ fractional inspired oxygen, *PaO*_*2*_ partial pressure of oxygen in arterial blood (mmHg).

Data are presented as median and interquartile range.

*Kruskal Wallis test.

### Safety outcomes

There are no significant differences in adverse event rates between groups. There were seven non-serious adverse events related to nebulized surfactant delivery, all due to a rise in peak pressure associated with heat and moisture exchangers (HME) clogging by surfactant, which were resolved by a change of HME in the ventilator circuit. There were no serious adverse events related to surfactant delivery (Table 5). Overall, 5 patients died, 1 from the control group and 4 from the surfactant group. Four patients died from refractory hypoxic respiratory failure, and one died from an intracerebral haemorrhage due to a brain tumour. The median time from last dose of surfactant delivery to death was 11 days (IQR 8.0–14.8 days).

**Table 5:** Safety outcomes.

AEs, SAEs and SUSARS	No. of events		No. of patients		RR (95% CI)	*p* value*
	Surfactant	Control	Surfactant	Control		
Total AEs	61/104 (59%)	43/104 (41%)	11/12 (92%)	8/8 (100%)	0.92 (0.77, 1.09)	1.000
Related to study drug	7/7 (100%)	0/7 (0%)	4/12 (33%)	0/8 (0%)	N/A	N/A
Related to study device	1 (100%)	0 (0%)	1/12 (8%)	0/8 (0%)	N/A	N/A
Related to study procedure	6/7 (86%)	1/7 (14%)	3/12 (25%)	1/8 (13%)	2.00 (0.25, 15.99)	0.619
Total SAEs	2/3 (67%)	1/3 (33%)	2/12 (17%)	1/8 (13%)	1.33 (0.14, 12.40)	1.000
Related to study drug (SAR)	0 (0%)	0 (0%)	0 (0%)	0 (0%)	N/A	N/A
Related to study drug and unexpected (SUSAR)	0 (0%)	0 (0%)	0 (0%)	0 (0%)	N/A	N/A
Pre-specified AE’s						
Any death	4/5 (80%)	1/5 (20%)	4/12 (33%)	1/8 (13%)	2.67 (0.36, 19.71)	0.063
Any cardiac arrest	0 (0%)	0 (0%)	0 (0%)	0 (0%)	N/A	N/A

*AE* adverse events, *SAE* serious adverse events, *SAR* serious adverse reaction, *SUSAR* suspected unexpected serious adverse reaction.

Data are presented as frequency and percentage of total, and risk ratios (RR) are reported with 95% confidence intervals.

*Fisher’s exact test.

### Surfactant phospholipid measurements

**Figure 4:**
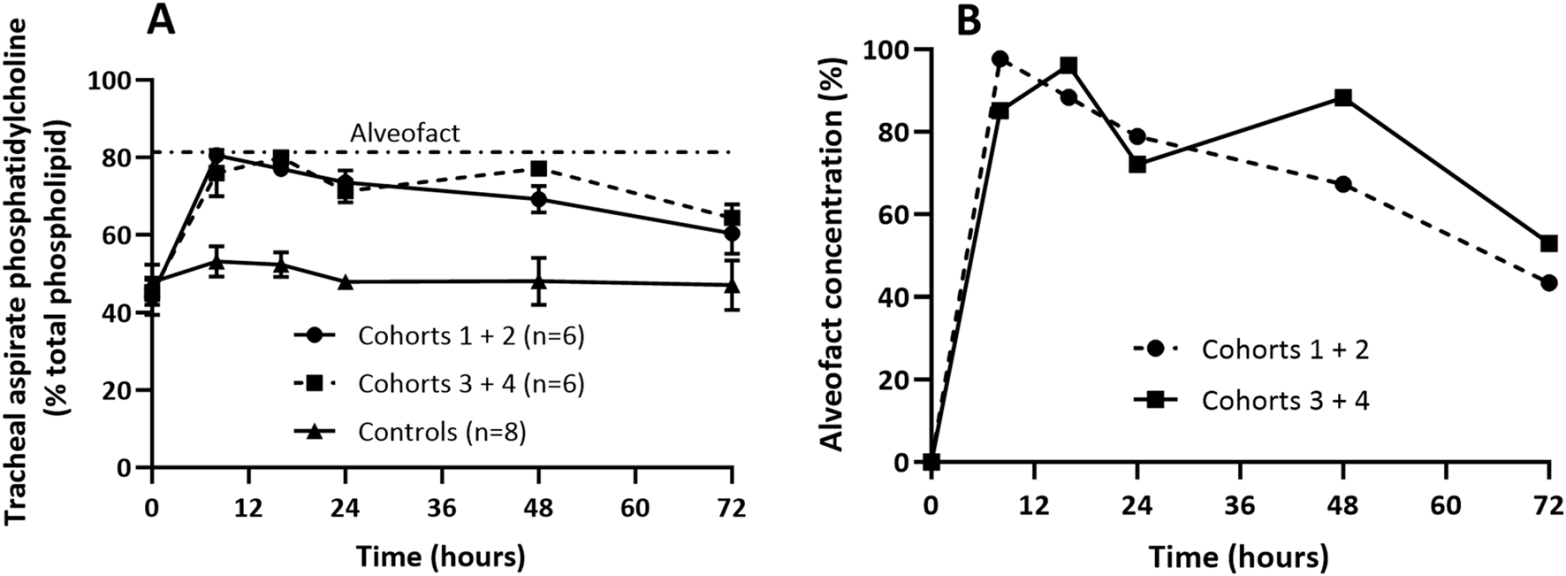
Phosphatidylcholine concentration in tracheal aspirates. (**A**) Total phosphatidylcholine concentration expressed as a percentage of total phospholipid. The horizontal line represents the percentage phosphatidylcholine analysed in Alveofact (81.4 ± 1.4%). (**B**) The contribution of Alveofact to tracheal aspirate phosphatidylcholine (%). Data are presented as mean ± standard error of mean.

Surfactant phosphatidylcholine (PC) was extracted from tracheal aspirate samples and analysed over time. The surfactant group able to augment total aspirate PC pool from 44.7% at t = 0 h to a maximum value of 80.2% after nebulisation. This was evident for both surfactant groups (cohort 1 + 2 and cohort 2 + 3). There was a steady decline in total PC concentration over time (Fig. 4A). We also calculated the proportional contribution of Alveofact to total tracheal aspirate PC. This indicated that aspirate PC post nebulization, relative to endogenous PC at t = 0 h, was almost exclusively derived from Alveofact at the earliest time points but declined to 48.1% by 72 h (Fig. 4B).

## Discussion

This is a pilot, randomised, unblinded, two-centre, controlled study evaluating the feasibility, efficacy, and safety of delivering nebulized surfactant for COVID-19 patients with acute severe hypoxic respiratory failure. Breath synchronised exogenous surfactant delivery is feasible, and surfactant phospholipid composition was augmented following nebulization^11^. The trial was not controlled for other medical interventions, which were provided at the physicians' discretion according to the local guidelines from emerging evidence. With TSC approval, the study dose and dosing times were modified after the completion of the second cohort following a DSMB review of surfactant phospholipid analysis which suggested that, although surfactant was adequately delivered, surfactant turnover was rapid with significant variation between patients^11^. Moreover, it took a median duration of 252 min to deliver the 10 vials. Consequently, for the subsequent cohorts, the dose was reduced to 5 vials (540 mg), nebulized more frequently (every 12 h until 48 h) and extended the duration of therapy to 72 h. This pilot, feasibility study showed that neither ten doses (1080 g) nor five, more frequently administered doses of nebulized surfactant (540 mg) were effective in improving oxygenation or ventilation index at 48 h after initiation of the intervention in severe COVID-19 patients when enrolled within 24 h of mechanical ventilation. As far as we know, this is the first randomised controlled trial to report on the use of nebulized surfactant in mechanically ventilated COVID-19 patients.

Although surfactant replacement is established and effective in neonatal respiratory distress syndrome (RDS) so far, no adult studies demonstrated mortality benefits^15,16,27^. Our study was different to those previous surfactant clinical trials conducted in the ARDS population. Firstly, the study was conducted in patients with a single disease entity (COVID-19) with the assumption that this would result in a more homogenous cohort of patients with primary lung disease, in contrast to other studies of surfactant in ARDS. Secondly, we used a novel breath actuated vibrating mesh nebulizer device to improve distal surfactant deposition. Porcine animal model studies using this device suggest that distal deposition (with > 50% deposition efficiency) is much higher than with traditional continuous nebulization^19^. Thirdly, we used a natural surfactant preparation, and these are generally preferred for the treatment of neonatal RDS due to their surfactant protein composition^28^. Fourthly, the tracheal aspirate PC analysis confirmed effective surfactant delivery in our study.

Recently, several case series and observational studies have used exogenous surfactant replacement in COVID-19 patients and reported improved clinical outcomes^29–33^. These studies varied from our RCT in several ways, including patient characteristics, surfactant preparation, dosage, and delivery methods. Both Busani et al. and Piva et al. used natural porcine surfactant on mechanically ventilated COVID-19 patients targeting those with low static lung compliance and delivered endotracheally via a suction catheter or direct bronchoscopy, respectively^29,30^. Although we did not pre-phenotype patients according to static compliance, the intervention group’s median static compliance was 42 cmH_2_O, slightly more compliant than these published case series. Our surfactant dosage was much lower compared with these published case series. However, we gave multiple doses to counteract the effect of surfactant inhibition, which was previously thought to be a significant issue.

In this a small, pilot, phase 2, feasibility, two-centre trial, we have shown that nebulized synchronised natural bovine surfactant is feasible to deliver without any significant adverse events. However, in mechanically ventilated COVID-19 patients, natural bovine exogenous surfactant at doses of 540 mg or 1080 mg did not improve oxygenation or other secondary outcomes. Furthermore, the surfactant group had a trend towards increased mortality. However, this finding could be attributed to disease severity rather than the intervention. Although nonsignificant, the surfactant group had higher APACHE-II and SOFA scores. Moreover, the surfactant group had a considerably greater neutrophil-to-lymphocyte ratio and CRP on admission (more than twofold), indicating increased disease severity^34,35^. The fact that the surfactant group spent less time in the hospital and on non-invasive respiratory support prior to mechanical ventilation may possibly indicate that the surfactant group had a more severe disease process than the control group. Furthermore, the temporal relationship between the intervention and mortality (with a median duration of 11 days) is an important consideration, along with the short half-life of Alveofact supports the notion that the intervention was unlikely to have contributed.

While it is biologically plausible that exogenous surfactant may improve clinical outcomes, or notwithstanding that surfactant therapy may be ineffective, this study raises several questions; (1) what is the correct patient group?; (2) what is the timing of surfactant treatment (early/late)?; (3) what the ideal surfactant for adults (natural/synthetic)?; (4) what the ideal dose (high dose/low dose) and delivery method (nebulized/intratracheal/bronchoscope)?; (5) What is the ideal interval between doses?; (6) why is there rapid surfactant turnover and how to minimise it?; (7) is high fractional inspired oxygen given to patients detrimental to surfactant function?.

The strength of the study include: (1) this is the first study to utilise a novel breath synchronized nebulizer device in combination with photo defined aperture plate vibration mesh technology to deliver exogenous surfactant in mechanically ventilated adult patients; and (2) demonstration of augmentation of surfactant phospholipids following nebulization followed by a rapid turnover of surfactant phospholipids^11^. However, the study also has several limitations: (1) Although we used a homogenous patient group (i.e., a single disease entity), there was significant clinical heterogeneity between patients, and we did not predefine the endogenous surfactant status pre-supplementation. There were significant variations between patients in clinical characteristics, thus our cohort may have included different clinical phenotypes due to discord between the degree of hypoxemia and ventilation mechanics^36^. (2) We did not control for the clinical management of patients including mechanical ventilation modes/settings, oxygen targets, use of prone positioning and other additional therapies such as augmented corticosteroids and pulmonary vasodilators. These interventions may have impacted on oxygenation and other clinical outcomes. (3) The primary outcome measurements of oxygenation change at 48 h showed substantial fluctuations throughout the day and can be influenced by other clinical manoeuvres. This could have influenced our findings, and a more complete summary of oxygenation during the day could have reduced intra-day variability. (4) Although there were no significant differences in the tidal volumes between the groups, both groups, in particular the surfactant group, had higher than the recommended (< 6 ml/kg IBW) targets^37^. While we worked towards the ARDS recommendations and guidelines, the higher tidal volumes reflect real-world practices and are comparable to previously published clinical trials of ARDS^38–40^. Furthermore, because this is a phase 2 pilot trial, the small number of patients recruited may not have been sufficient to detect significant clinical changes and outcomes.

In neonates, natural surfactant is the preferred choice for supplementation. Due to the large surface area and the need for larger quantities of exogenous surfactant, most adult studies on ARDS patients were conducted with synthetic surfactant preparations. Moreover, natural surfactants are more costly than synthetic preparations. We chose a well-established natural surfactant, which has shown proven benefit in neonatal RDS^41^. Traditionally, large quantities of exogenous surfactant have been delivered to prevent surfactant catabolism and inhibition. We gave 1080 mg for cohorts 1 + 2 and 540 mg for cohorts 3 + 4. Although this was much lower than previously published studies, it took nearly 4 h to deliver 10 vials (1080 mg). Our mechanistic analysis confirmed surfactant delivery and augmentation, but the half-life was rapid, suggesting there may be an increased breakdown either through hydrolysis following phospholipase activation pathways, oxidation due to alveolar hyperoxia and/or rapid uptake by activated alveolar macrophages. Scientific mechanistic studies evaluating these concepts to determine the fate of supplemented surfactant are urgently needed. Further analysis of surface function of the supplemented surfactant is underway which may provide further details on the in-vivo activity of supplemented surfactant.

## Conclusions

This phase 2 randomised controlled trial of natural nebulized surfactant using novel breath-synchronised delivery combined with a photo defined aperture plate (PDAP) vibrating mesh nebulizer in mechanically ventilated patients with severe COVID-19 is feasible and safe. However, the trial did not demonstrate improvement in oxygenation after 48 h of randomisation and does not support routine use of surfactant in severe COVID-19.

### Supplementary Information

Supplementary Information.

## Data Availability

The datasets used and analysed during the current study are available from the corresponding author on reasonable request.
