# Supplementary material for: A randomized controlled trial of nebulized surfactant for the treatment of severe COVID-19 in adults (COVSurf trial)
Source: Sci Rep. 2023 Nov 28;13:20946. doi: 10.1038/s41598-023-47672-x (PMC10684757; doi:10.1038/s41598-023-47672-x)
Supplement: Supplementary file 1 — Supplementary Information. [file 41598_2023_47672_MOESM1_ESM.docx]

A clinical trial of Nebulised surfactant for the treatment of severe COVID-19 in adults (COVSurf)

General Information

**Study details:** This is a pilot, dose exploratory, open-labelled, two-centre, randomized controlled study.

**Blinding (masking):**This is an open-labelled unblinded study.

**Numbers to be randomised (sample size):**The total sample size is 20 COVID-19 mechanically ventilated patients (12 intervention; 8 control).

**Trial registration:**ClinicalTrials.gov: [NCT04362059](http://clinicaltrials.gov/show/NCT04362059) (Registered 24 April 2020), EUDAMED number: CIV-GB-20-06-033328, EudraCT number: 2020-001886-35 (Registered 11 May 2020)

**Funding:** The trial is funded by Bill and Melinda Gates Foundation.

Rationale and background information

In December 2019, the Wuhan Municipal Health Committee identified an outbreak of viral pneumonia cases of unknown cause. Coronavirus RNA was quickly identified in some of these patients. This novel coronavirus has been designated SARS-CoV-2, and the disease caused by this virus has been designated COVID-19. This disease has now spread globally with a particularly high burden of disease in Europe and the USA.

Currently, there are no approved therapeutic agents available for coronaviruses. Initial epidemiological, radiographic, and pathological data from Wuhan in combination with basic cellular and molecular biologic data suggest that early surfactant dysfunction, may be a specific aspect of the high-morbidity coronaviruses SARS-CoV-1, MERS, and SARS-CoV-2. Accordingly, the possibility that early surfactant replacement could be beneficial in preventing progression of disease severity is especially compelling given the established safety profile of surfactant, which although officially indicated for neonates, has been used in many adult acute respiratory distress syndrome (ARDS) studies.

Lung surfactant is a lipoprotein complex comprising approximately 90% lipid, of which phosphatidylcholine (PC) is the principal component, with four specific surfactant proteins (SP-A, SP-B, SP-C, SP-D). Synthesis and secretion of the functional surfactant complex is uniquely confined to the type II cell (AT-II) of the alveolar epithelium (Goss 2013). Lung surfactant forms a monolayer covering the alveolar surface where it reduces the work of breathing by opposing surface tension forces and preventing alveolar collapse. The high surface pressure caused by surfactant is a major factor in minimizing the volume of the epithelial ling fluid of the alveoli and terminal bronchioles and preventing lung oedema. SP-B and SP-B promote formation of the surfactant layer at the air: liquid interface, while SP-A and SP-D are in the first line defense against microbial infection and bind a number of viruses including influenza.

The hypothesis behind the proposed trial of surfactant therapy for COVID-19 infected patients requiring ventilator support is that endogenous surfactant is dysfunctional. This could be due to decreased concentration of surfactant phospholipid and protein, altered surfactant phospholipid composition, surfactant protein proteolysis and/or oedema protein inhibition of surfactant surface tension function and/or oxidative inactivation of surfactant proteins. Variations of these dysfunctional mechanisms have been reported in a range of lung diseases, including cystic fibrosis and severe asthma (Postle 1999), and in child and adult patients with ARDS. Our studies of surfactant metabolism in adult ARDS patients showed altered percentage composition of surfactant PC, with decreased DPPC and increased surface tension-inactive unsaturated species, and decreased concentrations of both total PC and phosphatidylglycerol (PG) (Schmidt 2007, Dushianthan 2014).

The SARS-CoV-2 virus binds to the angiotensin converting enzyme-2 (ACE2) receptor (Hoffman 2020), which is preferentially expressed in the peripheral lung ATII cells (Qi 2020). Consequent viral infection of ATII cells could reduce cell number and impair the capacity of the lungs to synthesise and secrete surfactant. This, however, has not yet been demonstrated empirically in COVID-19 patients. If this is the case, then exogenous surfactant administration to the lungs is potential one treatment option to mitigate disease severity in these patients.

**Hypothesis: A deficiency of functionally intact surfactant contributes to the deterioration in pulmonary function in patients with severe COVID-19.**

References

Dushianthan A, Goss V, Cusack R, Grocott MP, Postle AD: Altered molecular specificity of surfactant phosphatidycholine synthesis in patients with acute respiratory distress syndrome. Respir Res 2014, 15:128.

Goss V, Hunt AN, Postle AD: Regulation of lung surfactant phospholipid synthesis and metabolism. Biochim Biophys Acta 2013, 1831:448-58.

Hoffmann M, Kleine-Weber H, Schroeder S, Kruger N, Herrler T, Erichsen S, Schiergens TS, Herrler G, Wu NH, Nitsche A, Muller MA, Drosten C, Pohlmann S: SARS-CoV-2 Cell Entry Depends on ACE2 and TMPRSS2 and Is Blocked by a Clinically Proven Protease Inhibitor. Cell 2020,181, 1:10.

Postle AD, Mander A, Reid KB, Wang JY, Wright SM, Moustaki M, Warner JO: Deficient hydrophilic lung surfactant proteins A and D with normal surfactant phospholipid molecular species in cystic fibrosis. Am J Respir Cell Mol Biol 1999, 20:90-8.

Qi F, Qian S, Zhang S, Zhang Z: Single cell RNA sequencing of 13 human tissues identify cell types and receptors of human coronaviruses. Biochem Biophys Res Commun 2020 <https://doi.org/10.1016/j.bbrc.2020.03.044>.

Schmidt R, Markart P, Ruppert C, Wygrecka M, Kuchenbuch T, Walmrath D, Seeger W, Guenther A: Time-dependent changes in pulmonary surfactant function and composition in acute respiratory distress syndrome due to pneumonia or aspiration. Respir Res 2007, 8:55.

Study goals and objectives

**Objectives:**SARS-Cov-2 virus preferentially binds to the Angiotensin Converting Enzyme 2 (ACE2) on alveolar epithelial type II cells, initiating an inflammatory response and tissue damage which may impair surfactant synthesis contributing to alveolar collapse, worsening hypoxia and leading to respiratory failure. The objective of this study is to evaluate the feasibility, safety and efficacy of nebulised surfactant in COVID-19 adult patients requiring mechanical ventilation for respiratory failure.

Study design

**Trial design:**This study is a dose-escalating randomized open-label clinical trial of 20 COVID-19 patients.

**Randomisation:**After informed assent, patients fulfilling inclusion criteria will be randomised to 3:2 for the treatment and control arms using an internet-based block randomization service (ALEA tool for clinical trials, FormsVision BV) in combination with electronic data collection. Randomisation will be done by the recruiting centre with a unique subject identifier specific to that centre.

**Participants:**This study is conducted in two centres: University Hospital Southampton and University College London Hospitals. Eligible participants are aged ≥18, hospitalised with COVID-19 (confirmed by PCR), who require endotracheal intubation and are enrolled within 24 hours of mechanical ventilation. For patients unable to consent, assent is obtained from a personal legal representative (PerLR) or professional legal representative (ProfLR) prior to enrolment. The following are exclusion criteria: imminent expected death within 24 hours; specific contraindications to surfactant administration (e.g. known allergy, pneumothorax, pulmonary hemorrhage); known or suspected pregnancy; stage 4 chronic kidney disease or requiring dialysis (i.e., eGFR < 30); liver failure (Child-Pugh Class C); anticipated transfer to another hospital, which is not a study site, within 72 hours; current or recent (within 1 month) participation in another study that, in the opinion of the investigator, would prevent enrollment for safety reasons; and declined consent or assent.

**Intervention and comparator:**Intervention: The study is based on an investigational drug/device combination product. The surfactant product is Bovactant (Alveofact®), a natural animal derived (bovine) lung surfactant formulated as a lyophilized powder in 108 mg vials and reconstituted to 45 mg/mL in buffer supplied in a prefilled syringe. It is isolated by lung lavage and, by weight, is a mixture of: phospholipid (75% phosphatidylcholine, 13% phosphatidylglycerol, 3% phosphatidylethanolamine, 1% phosphatidylinositol and 1% sphingomyelin), 5% cholesterol, 1% lipid-soluble surfactant-associated proteins (SP-B and SP-C), very low levels of free fatty acid, lyso-phosphatidylcholine, water and 0.3% calcium.

The Drug Delivery Device is the AeroFact-COVID™ nebulizer, an investigational device based on the Aerogen® Solo vibrating mesh nebulizer. The timing and escalation dosing plans for the surfactant are as follows. Cohort 1: Three patients will receive 10 vials (1080 mg) each of surfactant at dosing times of 0 hours, 8 hours and 24 hours. 2 controls with no placebo intervention. Cohort 2: Three patients will receive 10 vials (1080 mg) of surfactant at dosing times of 0 hours and 8 hours, and 30 vials (3240 mg) at a dosing time of 24 hours. 2 controls with no placebo intervention. Cohort 3: Three patients will receive 10 vials (1080 mg) of surfactant at a dosing time of 0 hours, and 30 vials (3240 mg) at dosing times of 8 hours and 24 hours. 2 controls with no placebo intervention. Cohort 4: Three patients will receive 30 (3240 mg) vials each of surfactant at dosing times of 0 hours, 8 hours and 24 hours. 2 controls. 2 controls with no placebo intervention.

The trial steering committee, advised by the data monitoring committee, will review trial progression and dose escalation/maintenance/reduction after each cohort is completed (48-hour primary outcome timepoint reached) based on available feasibility, adverse event, safety and efficacy data. The trial will not be discontinued on the basis of lack of efficacy. The trial may be stopped early on the basis of safety or feasibility concerns. Comparator: No placebo intervention. All participants will receive usual standard of care in accordance with the local policies for mechanically ventilated patients and all other treatments will be left to the discretion of the attending physician.

**Main outcomes:**The co-primary outcome is the improvement in oxygenation (PaO_2_/FiO_2_ ratio) and pulmonary ventilation (Ventilation Index (VI), where VI = [RR x PIP × PaCO_2_]/1000) at 48 hours after study initiation. The secondary outcomes include frequency and severity of adverse events (AEs), Adverse Device Effects (ADEs), Serious Adverse Events (SAEs) and Serious Adverse Device Events (SADEs), change in pulmonary compliance, change in positive end-expiratory pressure (PEEP) requirement of ventilatory support at 24 and 48 hours after study initiation, clinical improvement defined by time to one improvement point on the ordinal scale described in the WHO master protocol (2020) recorded while hospitalised, days of mechanical ventilation, mechanical ventilator free days (VFD) at day 21, length of intensive care unit stay, number of days hospitalised and mortality at day 28. Exploratory end points will include quantification of SARS-CoV-2 viral load from tracheal aspirates using PCR, surfactant dynamics (synthesis and turnover) and function (surface tension reduction) from deep tracheal aspirate samples (DTAS), surfactant phospholipid concentrations in plasma and DTAS, inflammatory markers (cellular and cytokine) in plasma and DTAS, and blood oxidative stress markers.

Statistical and data analysis

**Feasibility**

The adherence to per-protocol administration of Alveofact® will be by trial arm (ETI, NIV/CPAP, and by dosing regimen:

- Screening, enrolment and consenting of patients
- Number and proportion of participants with initial dose successfully administered according to protocol
- Number and proportion of participants with collection of relevant intermediate physiological outcomes and safety outcomes
- Number and proportion of participants with administration of subsequent doses according to protocol criteria
- Number and proportion of participants with collection of efficacy outcome data
- Number and proportion of participants who completed the study per protocol

**Safety**

The incidence and 95% Confidence Intervals of SAEs, and Grade 3 or 4 AEs will be summarized by trial arm and by randomization group over the first 24 hours, and throughout the study. The number and percentage of participants with AEs will be summarized by Medical Dictionary for Regulatory Activities (MedDRA) system organ class (SOC) and preferred term (PT). Additional summaries will present the number and percentage of participants with adverse events by intensity and by relationship to surfactant treatment. Summary statistics will be generated for raw laboratory safety tests, as well as for any changes from baseline, as deemed clinically appropriate.

**Efficacy**

The goal of this study is to assess the preliminary efficacy and safety of surfactant administration, identify an efficacy signal quickly (if present) to consider initiation of a larger randomised controlled clinical trial, and assess the potential impact of total dose on preliminary efficacy. 

The change in PaO_2_/FiO_2_ ratio relative to baseline at 24 hours after the last dose of surfactant and the change in Ventilation Index relative to baseline at 24 hours after the last dose of surfactant will be analysed separately.  For each endpoint, a test for a total dose-response effect using a model-based multiple contrast test (Bretz et al, 2005) will be performed.  Candidate dose-response models will include linear and Emax.  The change in PaO_2_/FiO_2_ and change in Ventilation Index may be transformed for analysis (e.g., log transformed) to better satisfy normality assumptions of model residuals.  The primary analysis will be done on patients with observed data at 24 hours (“completers”), though sensitivity analyses will also be performed if data are missing at 24 hours in an informative manner (e.g., due to death) where a non-parametric approach can accommodate missing data by assigning the worst rank.

The table below shows the power to show a significant dose response under varying assumed true dose response curves when using 12 matched controls.

|  | Relative change from baseline, on average | | | | |  | |
| --- | --- | --- | --- | --- | --- | --- | --- |
| CV | Control | Cohort 1 (30 total vials) | Cohort 2 (50 total vials) | Cohort 3 (70 total vials) | Cohort 4 (90 total vials) | Power to show dose response | |
| 20% | 0% | 0% | 0% | 0% | 0% | 0.05 | |
|  | 0% | 10% | 20% | 30% | 40% | 0.89 | |
|  | 0% | 25% | 25% | 25% | 25% | 0.82 | |
|  | 0% | 0% | 25% | 25% | 25% | 0.75 | |
|  | 0% | 0% | 0% | 30% | 30% | 0.72 | |
|  | 0% | 0% | 0% | 0% | 50% | 0.63 | |
| 25% | 0% | 0% | 0% | 0% | 0% | 0.05 | |
|  | 0% | 10% | 20% | 30% | 40% | 0.77 | |
|  | 0% | 25% | 25% | 25% | 25% | 0.66 | |
|  | 0% | 0% | 25% | 25% | 25% | 0.57 | |
|  | 0% | 0% | 0% | 30% | 30% | 0.54 | |
|  | 0% | 0% | 0% | 0% | 50% | 0.49 | |
| Based on 2000 simulations for each row. | | | | | | |  |

When the true residual CV is 20%, there is reasonable power to detect a dose response for either endpoint when the true increase is at least 20-30% in two or more cohorts.  There is limited power to detect a dose response when the effect is only in the highest cohort.  Also, when the true residual CV is larger, the power reduces substantially.  To reduce residual variability, the fitted model will also adjust for the baseline PaO_2_/FiO_2_ ratio or Ventilation Index as a proxy measure of baseline severity.  Significant dose response model fits can be utilized to estimate doses that provide clinically meaningful results and aid in planning for future studies.

Efficacy data will be continuously reviewed after each cohort to assess whether results are compelling enough to suggest initiation of a larger randomised controlled clinical trial.  Interim efficacy data will be analysed using a simple Bayesian adaptive approach.  After each cohort completes dosing plus 24 hours of follow-up, the posterior distributions of the mean change in PaO_2_/FiO_2_ ratio and the mean change in Ventilation Index will be determined under an assumed normal distribution with a non-informative prior.  The change in PaO_2_/FiO_2_ and change in Ventilation Index and may be transformed for analysis (e.g., log transformed) to better satisfy normality assumptions.  Interim efficacy will be declared for the purposes of further trial planning if there is relatively high posterior probability (e.g., > 80%) that EITHER the true mean change in PaO_2_/FiO_2_ OR mean change in Ventilation Index at 24 hours after last administration of surfactant “high” (e.g., ≥20%), either for an individual cohort or for all treated patients combined.

The following table shows the probability of observing at least 80% posterior probability that the true improvement is ≥ 20% on average for each endpoint.

| Relative change from baseline in each | Cumulative probability to observe at least 80% posterior probability that true change is ≥ 20% on average | | |
| --- | --- | --- | --- |
| group, on average | After 6 complete | After 9 complete | After 12 complete |
| 0% | 0.01 | 0.02 | 0.03 |
| 10% | 0.06 | 0.14 | 0.19 |
| 20% | 0.30 | 0.46 | 0.58 |
| 25% | 0.45 | 0.64 | 0.75 |
| 30% | 0.60 | 0.81 | 0.90 |
| 1000 simulations per row, assumes true CV = 20% | | | |

When there is no true improvement, there is < 5% probability to declare efficacy at an interim time point.  When the true improvement is 30% on average, there is 60% (81%) probability to declare efficacy at an interim time point after 6 (9) patients complete the study

Data management

The data will be managed with support from the Clinical Informatics Research Unit using ALEA eCRF® (FormsVision BV, Netherlands). This electronic CRF (eCRF) service for data collection and management provides comprehensive, user friendly forms service which can be used with a standard browser running on any computer connected to the internet. The system has been validated and has been certified by registered auditors to be in compliance with regulation, such as the FDA’s CFR 21 Part 11 and ICH GCP.

Anonymous data will be available for request from three months after publication of the article, to researchers who provide a completed Data Sharing request form that describes a methodologically sound proposal, for the purpose of the approved proposal and if appropriate, signed a Data Sharing Agreement. Proposals will be reviewed by the study team. Data will be shared once all parties have signed relevant data sharing documentation, covering the study team conditions for sharing and if required, an additional Data Sharing Agreement from Sponsor. Proposals should be directed to the chief investigator.

A data management plan will be in place once this protocol has been finalised.   At the end of the study and with sponsor approval, the database will be locked.

The statistician has access to the database who will undertake the data analysis following a statistical analysis plan. All data Management activities will be undertaken in accordance with CIRU SOPs that will be adhered to by all study staff.
